# Where’s the BIPOC Blueprint for Healthy Youth Development? The Role of Scientific Omissions in Our Struggle for Science Translation and Racial Equity in the United States

**DOI:** 10.1007/s10935-024-00771-5

**Published:** 2024-02-14

**Authors:** Valerie B. Shapiro, Nehal Eldeeb, Henrika McCoy, Miguel Trujillo, Tiffany M. Jones

**Affiliations:** 1 University of California Berkeley, Berkeley, CA USA; 2 University of Texas at Austin, Austin, TX USA; 3 University of Denver, Denver, CO USA; 4 Colorado State University, Fort Collins, CO USA

**Keywords:** Equity, BIPOC, Prevention, Healthy youth development, Evidence-based Programs, Unleashing the power of prevention

## Abstract

Prevention Science seeks to advance the prevention research and to translate scientific advances into the promotion of healthy development for all youth. Despite tremendous progress creating a robust evidence-base and set of translational tools, elaborations and expansions for equity are required. Our collective *errors of omission* as prevention researchers have left prevention practitioners and policy-makers without sufficient information to identify strategies that have been demonstrated to prevent behavioral health problems in young people who identify as Black, Indigenous, or other People of Color (BIPOC). We first describe the current shortcomings of available evidence, and then we call for individual and collective action to conceptualize equity-enhancing prevention, sample more inclusively, and improve analytic approaches such that we can truly promote the healthy development of all youth.

*Unleashing the Power of Prevention*, a discussion paper published with the National Academy of Medicine, articulated an inspirational vision and multi-method strategy for translating advances in prevention science to the promotion of healthy development for all youth (Hawkins et al., 2016). This catalyzing document articulated two overarching and intersecting goals: Reduce behavioral health problems in young people — and reduce embedded racial and socioeconomic disparities — by 20% within a decade. In order to leverage prevention science for social change, seven broad strategies to *Unleash the Power of Prevention* were articulated: (1) raise public awareness of behavioral health problems and the power of prevention, (2) increase funding for prevention initiatives, (3) establish criteria for preventive initiatives that are effective, sustainable, equity enhancing, and cost beneficial, (4) increase community capacity to assess and respond to their local prevention needs, (5) increase the infrastructure available to support the high-quality implementation of preventative initiatives, (6) increase the number of young people receiving effective preventive interventions, and (7) train and enable a prevention workforce (Shapiro & Bender, 2018). *This is a laudable call to action, which we wholeheartedly endorse*. We believe, however, that amidst this ambitious advocacy agenda, a deeper commitment to the promotion of behavioral health of Black, Indigenous and People of Color (BIPOC) communities is urgently needed.

We therefore focus this paper on the ways in which the call to *Unleash the Power of Prevention* can be leveraged to promote equity for Black, Indigenous, and other people of color through elaborations and expansions on prior omissions. In doing so, we first recognize that prevention science is not immune from participating in and inadvertently supporting structural racism, whereby interlocking systems of discrimination and violence disproportionately pattern the lives of BIPOC (Reskin, 2012). Structural racism is present in every institution and system in the United States, from our labor market (e.g., pay structure, working conditions, unemployment), school systems (e.g., tracking, discipline disproportionality, segregation), to our health systems (e.g., access to care and health insurance, discrimination in treatment) among many others (Reskin, 2012). Although research has shown that the results of these racist systems have their largest impact on Black people (Reskin, 2012; Singh et al., 2017), this paper uses the term BIPOC to acknowledge the violent histories of genocide of native peoples, slavery, Jim Crow, and mass incarceration (Alexander, 2010) that are at the foundation of the United States social contract. We recognize the complexity of the term BIPOC (Grady, 2020), and the nuances lost by grouping in this way (e.g., Latiné people who may or may not identify as a person of color); however, we find that BIPOC meets our goals of both fore fronting the specific experiences of Black and Indigenous people while taking into consideration what is shared with others who identify as people of color in the United States. This term also highlights the shared racialized experience of oppression of BIPOC, which separates them from people who identify as White in the U.S. context.

Throughout the manuscript, we (the authors) use the royal “we” to refer to the field of Prevention Scientists at large. We (the authors) recognize that many prevention scientists, largely scientists in the Global South and from low and middle income countries, as well as scientists of color and those with other multiply marginalized identities in high income countries, have been consistently calling for prevention science to center the voices and experiences of BIPOC, and have been working hard to advance equity within and through prevention science. Therefore, when we (the authors) use the royal “we” to refer to the work of prevention scientists in the United States, and those living within other economies that have benefited from forced labor and settler colonialism, we are referring to the ubiquitous nature of phenomenon being described, our collective responsibility for the current state of the field, and collective responsibility for reform – calling upon each and every prevention scientist to reflect, engage, and contribute to the promotion of behavioral health of BIPOC communities. This paper focuses on prevention in the United States, but carries our sincere hopes that scholars around the globe will point to our omissions, elaborate, and expand upon our writing.

## The Need for Equity Elaborations and Expansions

When the 2010 Affordable Care Act promised “a revitalized era for prevention” in the United States (Koh & Sebelius, 2010, p. 1296), it was well articulated that “without an explicit focus on equity, reform will leave millions of Americans behind” (Siegel & Nolan, 2009, p. 2401). Similarly, the action steps within each of the seven areas of *Unleashing the Power of Prevention* strove to decrease the *overall* rates of behavioral health problems in young people, although the plan to address the embedded racial and socioeconomic disparities was less explicitly articulated. This imbalance has the potential to perpetuate “the ‘national paradox of phenomenal scientific advancement and steady improvement in overall health status’ accompanied by ‘persistent, significant health inequities [that] exist for minority Americans’” (Koh et al., 2011, p. 1822).

Within the context of our laudable work as a field of prevention scientists, we reflect upon this imbalance as driven by *errors of omission*. In our conduct, curation, and creation of scientific advances, we often overlook the ways in which structural racism patterns the lives of BIPOC communities (Phelan & Link, 2015; Singh et al., 2017). Omissions in many of the framing and catalyzing documents of prevention science (e.g., Institute of Medicine seminal report on the Prevention of Mental Disorders) reflect and perpetuate a worldview that enables disparities to persist. For example, our early emphasis on “malleable” and “proximal” risk factors enabled us to demonstrate that the prevention of behavioral health problems is possible through research methods that were trusted by the medical establishment (Di Castri, 2024). As helpful as this was for gaining members and momentum, the downside was that it drew our attention to individual change mechanisms and away from systemic explanations and strategies.

The specific ways in which racism manifests for BIPOC are still often neglected in both our understanding of problems and in our strategies to prevent them. We have no reason to believe that oversights are deliberate or intentional, but *we dispute any sentiment that only deliberate omissions are problematic*. Innocuous practices of omission are ways in which hierarchies of dominance can inadvertently be maintained. As described by Jones (2000) at the turn of the century, institutional racism occurs when seemingly innocuous policies and practices result in disproportionate harm to particular racial and ethnic groups. Institutional racism doesn’t require intent but is inherent in its outcome; it is codified in custom, practice, or law, and is often indicated by *inactions* in the face of need. Twenty years later, the racial reckoning of 2020 in the United States has broadened awareness within our communities of scholarship, practice, and policy-making that racism need not be conscious or active in order for our ideals for the healthy development of all youth to be thwarted by the vast omissions we make. Without explicit attention to the experiences of BIPOC and equity, we run the risk of using available science to improve the health of some young people, shifting population level averages, without catalyzing change in the larger, social contexts in which young people develop disparately (Biglan et al., 2012, 2023; Ginwright et al., 2005).

## Our Collective Errors of Omission

Calls for *health equity* insist that obstacles be removed and resources be provided so that all people have a fair and just opportunity for health (Braveman et al., 2018; Boyd et al., 2023). Like others (e.g., Alvidrez et al., 2019; Barrera et al., 2017; Biglan et al., 2023; Ford & Airhihenbuwa, 2010; Marsiglia & Booth, 2015; Jagers et al., 2019; Stanley et al., 2020), we argue that these obstacles are socially constructed, with some constructed through our very efforts to advance prevention science. We, in the most grand and ubiquitous sense of the term, make omissions at every phase of the prevention research cycle. First, we fail to fully conceptualize racism and inequality in our definitions and estimates of the prevalence of problems. In the literal act of counting, some people are rendered more or less visible based on how they are positioned within society (Sacks, 2018). For example, Black people in the United States are systematically undertreated for pain because their complaints of pain are taken less seriously (Hoffman et al., 2016). The problems people experience can be differentially tallied based on how they are over-looked, over-surveilled, or plainly misunderstood through the lens of the counter. Further, the race of individuals is often used as a proxy for racialized experiences and individual experiences with racism; this practice risks a mischaracterization of the problem, misspecification of explanatory models, misinterpretation of results, and constrains the range of possibilities for intervention (Jones et al., 1991; Krieger, 2000).

Structural racism is often not measured in epidemiological research (Adkins-Jackson et al., 2022). This creates a body of literature where macro forces continue to be disproportionately omitted as explanatory pathways and intervention targets. Even when included, single dimension measures of racism, such as housing discrimination, disparate rates of incarceration, or neighborhood segregation do not sufficiently capture the ways these systems are interwoven and mutually reinforcing (Adkins-Jackson et al., 2022; Reskin, 2012). For example, people from BIPOC communities in the United States are imprisoned at alarming rates, resulting in a prison population where Black people are incarcerated at 3.5 times the rate of White people (Minton & Zeng, 2021). In conceptualizing this problem, it would be an error to obscure or omit the institutional mechanisms (e.g., disproportionate discipline, tracking) –broadly articulated as the school to prison pipeline– that illuminate previously under-acknowledged etiologies: Black students being expelled from school at rates 2 to 3 times their White peers (Skiba et al., 2011). Our collective discovery that well-designed and well-implemented social skills curricula can effectively improve youth behavior is an important advance that should not be disregarded, particularly relative to the widespread use of ineffective social skills curricula, but this strategy alone is unlikely to address factors that ultimately explain more of the variance in youth development. It also continues to place blame and burden on youth and the adults that socialize them, rather than acknowledge the structural racism that drives the system and its responses. Thus, this calls for additional approaches to theorization in prevention science (e.g., ecosocial theory [Krieger, 2012], Indigenous stress coping model [Walters & Simoni, 2002], cultural wealth [Yosso, 2016]) that starts at conceptualization of the problem we seek to prevent and relies, to a greater extent, on diverse disciplinary knowledges and ways of knowing for designing, implementing, and scaling solutions.

Even within our familiar risk and protective factor framework, often leveraged at the individual level, we do not adequately include experiences with micro-aggressions (i.e. “subtle insults (verbal, nonverbal, and/or visual) directed toward racial minorities, often automatically or unconsciously” [Solarzano et al., 2000 quoted in Wong et al., 2014]), discrimination, and racial trauma (e.g., Clark, 2001; Comas-Diaz et al., 2019; Trent et al., 2019) as endemic (i.e., universal within a subgroup) risk factors (Dixson & Rousseau, 2014) for poor behavioral health outcomes, nor positive racial and ethnic identity and related constructs as potential protective factors (e.g., Rivas-Drake et al., 2014; Spencer et al., 1991). Many meta-analytic reviews have documented the deleterious effects of microaggressions on a range of physical and mental health outcomes for BIPOC individuals (Choi et al., 2022; Costa et al., 2023; Wong et al., 2014). Omitting factors crucial to the development of BIPOC youth continues to privilege Whiteness and the facets of identity most relevant to White youth. For example, a review of 47 studies found that positive racial and ethnic identity is associated with improved academic achievement (Miller-Cotto et al., 2016). Further, positive racial and ethnic identity has been linked to adaptive psychosocial and health outcomes (Rivas-Drake et al., 2014) and found to be protective against experiences of discrimination by teachers (Thomas et al., 2009). Yet, the development of positive racial and ethnic identity is often not included in population-wide prevention approaches.

We further fail to articulate the ways in which bias is likely to enter the process of prioritizing the risk and protective factors that program developers target for change. For example, we largely ignore research that has shown that within-child characteristics are often used to explain the behavioral health problems of Black youth, whereas external factors are often used to explain the behavioral health problems of White youth, and judgments as to whether risk and protective factors are malleable with a given context are subject to similar racialized expectations and interpretations (McCoy & Pearson, 2019). This dynamic is particularly damaging for Black youth, for whom damaging deficit narratives perpetuate a cycle of marginalization (Baldridge, 2014). We fail to acknowledge that many marginalized youth are likely to receive universal interventions that do not recognize trauma, terror, racism, and other important correlates of their behavioral health (Marsiglia & Booth, 2015; Scott & McCoy, 2018; McCoy et al., 2016), and inversely, that many system-involved youth who could benefit from universal prevention practices, do not have access to such programs (McCoy & McKay, 2006).

There are many examples of scholars who have developed theories and guidance towards the development of culturally affirming interventions, stating that cultural adaptation is a necessity when striving towards the desire for universal interventions (e.g., Nation et al., 2003; Barrera et al., 2011; Buckley et al., 2023; Gaylord-Harden et al., 2012; Jones & Neblett, 2016). Yet many of us continue to omit this guidance, accepting as “universal” that which has been demonstrated to be effective with predominantly White samples. In starker terms, years of scientific funding to predominantly White researchers (Ginther et al., 2011), testing interventions with predominantly White samples (e.g., LaRoche & Christopher, 2008; Pacific Northwest Evidence-based Practice Center, 2019; Rowe & Trickett, 2018), has led us to rely on a remarkably robust, but admittedly narrow, scientific knowledge base. Our current focus on “scaling up” (i.e., promoting broad access to) existing preventive interventions, with the authentic and heartfelt aspiration to benefit *all* youth, overlooks gaps in theory, research, policy, and practice that specifically pertain to BIPOC youth.

In our efforts to disseminate prevention research, we first need to recognize that we actively reproduce racist systems through our contemporary contributions to the scientific literature at each phase of the Mrazek & Haggerty (1994) prevention research cycle, and that as contemporary prevention scientists, we have the opportunity to deconstruct or reconstruct the racial hierarchy every day while progressing our contributions to the field. For example, reflecting and confronting our personal biases, whether conscious or unconscious, is a foundational scientific practice. We each need to rethink which articles we consume, share, teach, and cite. We need to reconsider to whom we look for expertise and advice. We need to reconstitute our research and collaboration teams and position members to contribute diverse perspectives. These are all ways we can alter our approach to this work. With these activities incorporated into the regular routines of prevention research, we are *then* better positioned to think critically about our theoretical and methodological choices, and how these choices can reduce reflexive omissions and advance the science of prevention.

Second, we need to disavow ourselves of the belief that our science is *complete* and *ready for translation* when so many ecological contexts and people have been omitted from and marginalized within our evidence-base (Boyd et al., 2023). In the pages that follow, we delve into these two points. First, we illustrate one example of the problems caused by our omissions, and second, we consider opportunities for building upon the important work of our predecessors, and progressing the conduct of the prevention research that is required of us today.

## Where is the BIPOC Blueprint for Healthy Youth Development?

Like our colleagues attempting to scale up evidence-based interventions more broadly, persistent legacies of White supremacy have shaped our current lists (i.e., curated repositories) of effective prevention programs. In making this point, we do not intend to disparage the ideals and contributions of science-based prevention, the promise of curated lists of rigorously tested programs, or the programs currently on those lists which have demonstrated positive effects on the behavioral health of young people. *Fundamentally, we believe that science-based prevention can and should be a pathway to equity*. *Yet, we also believe that without explicit scrutiny and action to advance racial equity, scientific processes alone will not be able to overcome the racist societal systems in which they function*. The history of distrust of science by BIPOC communities in the United States is well earned, and has contributed to the historical trauma of Black people (Bajaj & Stanford, 2021). Thus, scientists, of which prevention scientists are no exception, have a distinct obligation to provide (and ideally, collaboratively generate and communicate) evidence that prevention efforts can benefit BIPOC people.

Notably, many of the prevention programs on such lists were designed with the underlying intention to be culture-blind, yet they have embedded an assumption of White hegemonic neutrality. They then evolved to be rhetorically inclusive (i.e., illustratively including names and images from people presumed not to be White so as to encourage adoption in diverse communities), but largely have not been structurally situated within or adapted to norms or beliefs beyond White cultural contexts (Castro et al., 2004). As written by Kumpfer et al. (2002),

Most universal prevention programs are generic programs developed for popular American culture or youth culture, which is heavily influenced by White, middle class values. Professional training has stressed ‘the melting pot’ model of American culture, resulting in few culturally-specific models (McGoldrick & Giordano, 1996). The theoretical constructs, definitions of protective or risk factors, appropriate intervention strategies, and research evaluation strategies have all been influenced by mainstream American values (Turner, 2000). Commercial developers seek to develop generic programs culturally acceptable by diverse families; thus, making their products widely marketable (p. 242).

The Blueprints for Healthy Youth Development is a registry of “experimentally proven programs” intended to guide decision-makers toward effective prevention practice (Mihalic & Elliott, 2015). It is one of 18 U.S.-focused, web-based registries that assign an intervention effectiveness rating to strategies for improving community behavioral health (Hirsch et al., 2023). We examine the Blueprints list because we think it is the *best* available menu of evidence-based prevention programs. Distinction as a “model” program typically requires two randomized controlled trials (RCTs), with sustained positive (at least 12 months after the program ends) and no iatrogenic effects. Given the rigor of the registry, it is the virtual place where communities conducting science-based prevention are advised to visit to select an effective prevention program for use. It is unclear, however, who is included in the studies buttressing the evidence-base of Model programs and whether some subgroups benefit from promoted interventions more than others. Looking at the Blueprints registry from the perspective of a research-user who hopes to increase the number of young people in their community receiving effective preventive interventions, what information can be gleaned about effective prevention for BIPOC youth?

The Blueprints website uses three strategies to enable consideration of health equity factors: target group filters, Fact Sheets, and a designated section on an individual intervention page which describes “Racial/Ethnicity/Gender Details”. When website visitors use target group filters (e.g., “African American” “American Indian / Alaskan” “Asian” “Caucasian/White” “Hispanic or Latino”) embedded in the Blueprints website, there is currently (2023) only one Model program (GenerationPMTO) listed as efficacious with Hispanic or Latino youth, no Model programs listed as efficacious with African American youth, no Model program listed as efficacious with Asian youth, and no Model programs listed as efficacious with American Indian/Alaskan youth. Beyond the target group filters, one could examine Blueprints Fact Sheets where 94% of Model programs are described as applicable to “Race/Ethnicity: All.” The final program (ParentCorps) is missing the entire category of Race/Ethnicity from its factsheet. This leaves the prospective research-user in quite a precarious position. Are they to believe only one prevention program has high-quality evidence for use with (some) BIPOC youth, or that only one prevention program *lacks* high-quality evidence for use with some BIPOC youth?

With the image in mind of a particularly persistent community prevention coordinator, we used the expandable menu containing “Race/Ethnicity/Gender Details” as described by Hirsch and colleagues (2023). One Model program, for example, had some nuance on this expandable menu. It stated “significant moderated effects showed that the intervention had greater benefits for non-Hispanic White families than Hispanic families” (Sandler et al., 2020). If the research-consumer is then able to access and appraise the primary research article, the reader would find a study that asks important questions, uses appropriate methods, and concludes “many of these moderated effects showed positive benefits for non-Hispanic White families but not for Hispanic families. The findings indicate… the need for further adaptations to make the program effective for Hispanic parents.” This adds another point of confusion for science-based decision making; this Model program is listed as “Race/Ethnicity: All” while surreptitiously citing a study that indicates non-significant effects for Hispanic families, that in fact, may widen disparities. This kind of error likely contributes to the legacies of distrust that shape the relevance and utility of curated lists of effective prevention practice in service of BIPOC youth.

To be clear, we have no intention of shaming those who promote and maintain the Blueprints Registry. *Blueprints is a powerful tool for evidence-based prevention practitioners*. Although there is room for modest improvement in the registries’ filters and factsheets, this problem can readily be remedied. Registries, however, can only make use of the research that exists (Hirsch et al., 2023). To the extent that there are underlying scientific omissions, these problems are more fundamental and it will take much longer to achieve solutions.

To this end, a recent review (Shapiro et al., 2023) of the Blueprints registry took a closer look at the science *underlying* the Model programs in the registry. Model (inclusive of “Model plus”) programs were selected by nature of their evidentiary distinction. In April 2020, authors identified 17 Model programs that were classified as such based on evidence from 44 Blueprints Certified studies, described in 62 peer-reviewed papers, published from 1973 to 2018 (median year = 2006). This review focused on the 40 Blueprints Certified Studies that were conducted in the United States, predicated on the notion that race is constructed and constrained differently across national contexts (Buckley et al., 2023; Flanagin et al., 2021), and that Certified Studies are what give the programs their evidentiary distinction. Of the 40 U.S. studies referred to by the Blueprints registry, 39 (97.5%) described the race/ethnicity of their sample. This compares favorably to descriptions about race (77%) and ethnicity (64%) provided by Buckley and colleagues (2023), who examined one study from each Blueprints program (2010–2021), rather than just Certified Studies from Model Programs. While most Certified Studies acknowledged race, the complexities of this social construct remained under examined. While 46% of studies used self-reported race, the majority of researchers used a third-party source to ascribe race to participants. Nearly a third of studies divided participants into only two racial groups (e.g., “White / non-White”), perpetuating racial hierarchies that hold White people as a comparison point for all others, and limiting the utility of the research for anyone who is grouped into the heterogeneous “Non-White” group. Only 30% of studies reported the presence of multi-racial individuals (*n* = 12), grouped together with sparsely represented mono-racial groups as “other” (i.e., not a racial group otherwise described; *n* = 7), or separately as “mixed ethnic heritage” (*n* = 2), “multiracial” (*n* = 1), “mixed race/ethnicity” (*n* = 1), or “multiple ethnic backgrounds” (*n* = 1). This implies that multi-racial people continue to be ignored, sorted in accordance to the non-transparent worldviews of the author, and/or excluded from any meaningful analysis. This practice may seem benign in comparison to historic racial purity doctrines in the United States (e.g., partus sequitur ventrem, racial hygiene, blood quantum), whereas a person is included or excluded based on short-hand schemas devised for the convenience or benefit of the sorter, and to the detriment of those being sorted. Yet, in our modern practice of research, the power to classify continues to distribute burden and benefit in accordance to the preferences of the people writing the protocols.

In the review by Shapiro and colleagues (2023), the racial/ethnic distribution of the study samples were categorized by researchers as having “no racial predominance” if no single race comprised over 50% of the study sample, a “modest racial predominance” if a single race comprised more than 60% of the sample, and an “extreme racial predominance” if a single race compromised more than 80% of the sample. This review found that 11 (28%) certified studies reported no racial predominance, 24 (60%) had a modest White predominance (13 of which had an extreme White predominance), three had an extreme Black predominance (8%), one study had a modest Hispanic predominance (3%), and one certified study did not describe the race of their sample (3%). Using the three certified studies that had an extreme Black predominance as an example, we find studies of Functional Family Therapy (Gottfredson et al., 2018), the Nurse-Family Partnership (Kitzman et al., 1997; Olds et al., 2004), and ParentCorps (Brotman et al., 2013, 2016). In this limited number of studies, overall study findings are likely to apply to Black youth (to the extent that any sample is expected to generalize beyond the historical moment, and the sample was sufficiently large and diverse in regard to other, intersecting identity characteristics). In contrast to other reviews, we think it was important for authors to look at the representation *within* a study, rather than an entire body of literature, in order to understand to whom the evidence generated applies. A total of 14 studies, exploring a total of 10 programs, had a percentage of “Black” or “African American” youth in their samples that met or exceeded 13.6% of the study sample, a percentage which could be considered proportional to Black youth in the United States (U.S. Census Bureau, 2021). The claims generated through the analysis of the full sample in these studies may, or may not, apply to Black youth; this inference would require that race be incorporated into the analysis.

Of all Blueprints Model programs in this review (*n* = 17), 10 programs (59%) were buttressed by certified studies that used race as a variable in the analysis. Five of these programs were supported by studies that only stated that they used race as a covariate, some of which did not represent race in any data table or otherwise report or interpret the role of race in their findings (e.g., Botvin et al., 2006; Letourneau et al., 2009). Some controlled for race at the individual level (Letourneau et al., 2009), while others controlled for demographics at the implementation site (e.g., Botvin et al., 2006). Some ultimately dropped covariates due to collinearity (e.g., Letourneau et al., 2009). Of the 17 Model Blueprints programs, only 5 (29%) were buttressed by studies that expressed intentions to examine treatment effects by race. Because race did not directly predict outcomes independent of other covariates, one study did not pursue an interaction (Olds et al., 1997). Another stated that there was “no evidence that participant ethnicity moderated the intervention” without any specification of method or display of data further explicating this claim, while also writing that “the sample was relatively homogeneous with regard to ethnicity and socioeconomic status, suggesting that care should be taken in generalizing the results to move diverse populations” (Stice et al., 2011, p.504).

This leaves three Model programs (17%) whose Model distinction status was buttressed by studies that ultimately examined treatment effects by race. Here, we describe findings from the Shapiro and colleagues (2023) review, using terms to describe the samples that reflect the original language of these studies. They report:

A study examining Multisystemic Treatment (MST) evaluated the effects of several potential moderators, including race (“White” / “African American”), on posttreatment arrests by examining the cross-product term of the treatment group and the moderating variable, entered last in a sequential regression model (Borduin et al., 1995). A non-significant change in R^2^ for the cross-product term was interpreted to mean that MST was “equally effective with youths of different… ethnic backgrounds” (Borduin et al., 1995, p. 576). ParentCorps and Early College High School Initiative (ECHS) reported significant intervention effects by racial group. The ParentCorps study (Brotman et al., 2011) observed no primary outcomes (neither effective parenting practices nor child behavior problems) moderated by race (families characterized as “Black” or “Latino”), but did observe moderation of a secondary outcome (parental involvement), such that there was only an effect for “Black” families. In one ECHS study (Haxton et al., 2016), findings are interpreted by the authors as reducing a disparity (i.e., there was a stronger effect for “minority students” than for “nonminority students” on “post-secondary degree attainment”, having defined “minority students” as “non-White”) on one of the five outcomes examined. In a second ECHS study (Edmunds et al., 2017), however, the intervention exacerbated a disparity (i.e., there was a stronger effect for “not underrepresented” than for “underrepresented minority students” on “college credits accrued” and “percentage of students who received any postsecondary credential,” having defined “underrepresented minority students” as students who identify as “African American, Hispanic/Latino, or Native American”).

Reviewing this (very small) selection of intervention studies begins to illustrate some of the complexities of analyzing subgroup effects in ways that advance equity in research and inform practice and policy decisions. Classic methodological debates are evoked when considering the disaggregation and analysis of preventive intervention effects by race (Kaufman & Cooper, 2001). How should race be determined? How should race be bounded such that it represents something meaningful, given within-group variance in broadly constructed categories that are not rank-ordered nor mutually exclusive? The ways in which race variables are coded and comparisons are drawn has implications for the size, significance, and interpretation of the intervention effects, and readily, if not transparently, embed the researcher’s worldview in their research. In cases where there is racial diversity within samples, the subsamples are often quite small, posing threats to validity and generalizability. Small samples often do not allow adequate power to test for race moderation, yet under-powered null interactions are often interpreted to mean that there are no differences in intervention effects by race. This well intended effort, to actually analyze race, inadvertently biases the interpretation of intervention studies (with main effects and some racial diversity) to be considered “universal” – despite the fact that there is no specific evidence of effectiveness for BIPOC youth. Subgroup analyses that analyze within group effectiveness are the strongest evidence to support claims of effectiveness in specific communities. Further, this focus on *race* only considers one of many variables (e.g., primary language, national origin, migration history) that might contribute to generalizability and/or implementation success. This synthesis merely begins to exemplify the ways in which we fall short of our own Standards of Evidence for Efficacy, Effectiveness, and Scale-up (Gottfredson et al., 2015), which require the demonstration of intervention effects across population subgroups as a prerequisite for scaling up evidence-based interventions.

## Equity-Enhancing Prevention

There are many ways in which we could strive to overcome our historic omissions in ways that help us not just feel better, but actually do better to counteract the forces of racism. Returning to the call to *Unleash the Power of Prevention*, we have been given an impetus to establish consensus criteria for *equity-enhancing prevention* (Hawkins et al., 2015). Hirsch and colleagues (2023) consider the ways in which notions of equity could be embedded into registries, with interventions rated for their health equity impact. Boyd and colleagues (2023) envision narrative style healthy equity impact statements. Yet, for many of us, all of this begins with achieving clarity about our own conceptualization of equity-enhancing prevention (Shapiro et al., 2023). For example, a *centering* approach foregrounds questions of relevance to the well-being of BIPOC youth, rendering comparisons to other groups a distraction from that purpose. Under this aim, a study is designed to test whether an intervention is effective for a marginalized group with the explicit goal of improving the group’s outcomes (Duran & Pérez-Stable, 2019). A *reducing* approach prioritizes the observation and reduction of disparate care and outcomes between structurally advantaged and disadvantaged groups (see Pérez-Stable & Rodriquez, 2023). These studies are designed to detect interactions and claim whether an intervention reduced or exacerbated a between-group disparity. A *protecting* approach prioritizes the observation of equality, seeking to determine whether all groups benefit, and to the same extent. These studies explore the benefits of our approaches, and interpret the lack of significant race-by-treatment effect findings as good for all people, although they also maintain our existing disproportionate resource distribution and disease burden (see Frohlich & Potvin, 2008). A *promoting* approach assesses the extent to which obstacles can be removed, and resources be provided, so all youth can benefit in ways that maximize their behavioral health (see Boyd et al., 2023). These studies are designed in the spirit of *targeted universalism* (Powell et al., 2019), whereas the goals are the same for all groups, but the strategies to achieve the goals are targeted (i.e., differentiated in accordance to diverse cultures, contexts, and conditions). Prevention science offers us the tools to do any and all of this, with a role for each of us. If we put in the necessary and collaborative effort to overcome the legacies of mistrust, we could pursue the requisite evidence to eventually have a Blueprints registry with relevance and utility to and for BIPOC communities.

Earning trust often requires becoming vulnerable. Those of us privileged enough to have a secure appointment at a research institution need to review the processes of conformity that we have accepted and perpetuated in order to be there. We need to recognize/affirm that our scholarly capacity to take risks and challenge conventional wisdom is “stymied by an entire ecosystem of legitimacy and validation built upon a profoundly colonial epistemology nested within a neoliberal economy” (Park et al., 2020, p.447). We lament that research knowledge is not frequently used in practice and policy settings (Davies & Nutley, 2008), but if we want our scientific contributions to be received differently, we need to create them differently (e.g., with participation of those closest to the problem). We need to recommit ourselves as change agents within our institutional homes (e.g., universities) and to the institutions we serve (e.g., the National Institutes of Health, Society for Prevention Research) as scientific advisors, editors, and conference organizers. We must be thoughtful peer-reviewers who distinguish between *rigor* (i.e., the quality of being extremely thorough) and *reification* (i.e., believing that humanly created social processes are absolute things) in our own work and in the appraisal of the work of others. To have evidence that is actually *used* to improve the lives of young people, we need to foreground an equity-enhancing prevention agenda that starts with equity-enabling scientific processes. We reiterate here Dr. McCoy’s (2020) reminder of our collective responsibility to include and amplify the voices of informed and engaged BIPOC researchers, and to ensure our research processes, from conceptualization to interpretation, are “filtered through eyes that do not benefit from white privilege” (p. 472).

## Conclusion

In summary, it appears that when evidence-users try to ascertain whether a Model Blueprints program has been demonstrated to be effective with young people like those in the community in which they live or work, they may not be able to make this determination. Although U.S.-based studies buttressing the Blueprints Model prevention programs do typically describe the race and/or ethnic composition of their study sample, the ways in which race is conceptualized, measured, and used could be improved. The studies buttressing Blueprints Model program distinctions have been conducted on predominantly White samples, although three studies centered Black youth. Very few studies reported interactions or subgroup effects by race. This provides little information as to whether rigorously tested prevention programs perpetuate, sustain, or remediate racial and ethnic disparities in behavioral health and outcomes, or more broadly, promote the behavioral health of BIPOC youth. Viewed through several different approaches, there are exceedingly few Blueprints programs that showed specific evidence of effectiveness for BIPOC communities, and this is likely the case across other similar registries. Many of us are bystanders as these critical questions are left unanswered among calls for programs to be implemented in BIPOC communities without evidence. We, collectively, must admit that the use of these programs with BIPOC communities is experimental, rather than translational, and in many cases, the experiments are being conducted without systematic data collection or any robust analysis. This quite reasonably perpetuates mistrust of our work within BIPOC communities.

Without explicit evidence of efficacy for BIPOC youth, the justification for “scaling up” programs requires maintaining an assumption of White universalism and the acceptance of avoidable scientific errors. On the other hand, we recognize that *withholding* effective prevention programs from understudied populations could also adversely impact the behavioral health of BIPOC youth. Therefore, we join the call of Aarons and colleagues (2017) for researchers to work as urgently on scaling *out* effective prevention programs (i.e., adapting to novel populations or delivery systems) as on scaling them up. When doing so, we must allocate adequate funding for the formation of authentic and long-lasting research-practice partnerships, and for the protection of human subjects, data collection, analysis, and dissemination as an acknowledgment that this work is *experimental* rather than *translational* – it requires the informed participation of intended beneficiaries and all of the safeguards of scientific ethics in order to avoid unintended harms. Our research funders, journal editors, and repository curators need to actively support, circulate, and track scale out efforts (Alvidrez et al., 2019). In our work, we must interrogate power *within* prevention, as part and parcel to our efforts to Unleash the Power *of* Prevention – to reduce disparities in the behavioral health of young people.

In sum, prevention coalition members should be able to efficiently identify a program that has been studied and demonstrated to prevent behavioral health problems in young people who identify as Black, Indigenous, or other People of Color. If a program has not yet been studied and demonstrated to be effective for BIPOC youth, we should develop protocols for intervention modification, use, testing, and incorporation into our knowledge base. In addition, we need to expand our scientific inquiries to processes that do not emanate from what has shown to be effective for White populations, but alternatively builds and amplifies a knowledge base that centers BIPOC experiences. Note, we are not the first to suggest either of these approaches (see Alvidrez et al., 2019, Barrera et al., 2017; Ford & Airhihenbuwa, 2010; Marsiglia & Booth, 2015; Jagers et al., 2019; Stanley et al., 2020); we are simply reiterating that the need remains unmet. If we continue to under-theorize, under-sample, under-analyze, or blatantly ignore race and the impact of racism in our studies, we will continue to bemoan the failures of communities to use science to inform their practice, without acknowledging that it was us who abdicated our responsibility, as researchers, to provide trustworthy evidence central to local prevention planning and the healthy youth development.
